# Mechanisms controlling human head stabilization during random rotational perturbations in the horizontal plane revisited

**DOI:** 10.14814/phy2.12745

**Published:** 2016-05-24

**Authors:** Ann‐Katrin Stensdotter, Morten DinhoffPedersen, Ingebrigt Meisingset, Ottar Vasseljen, Øyvind Stavdahl

**Affiliations:** ^1^ Faculty of Health and Social Sciences, PhysiotherapyThe Norwegian University of Science and TechnologyNTNUTrondheimNorway; ^2^ Department of Public Health and General PracticeFaculty of MedicineThe Norwegian University of Science and TechnologyNTNUTrondheimNorway; ^3^ Department of Engineering CyberneticsFaculty of Information Technology Mathematics and Electrical EngineeringThe Norwegian University of Science and TechnologyNTNUTrondheimNorway

**Keywords:** analysis methods, motor control, neck

## Abstract

This study repeats the experimental protocol for investigation of head stabilization in healthy humans, described by Keshner and Peterson (1995) but with a modification of the analysis. Head movements were considered with respect to the room instead of relative to the trunk. The aim was to investigate the approximate contribution of reflex and voluntary control across perturbing frequencies and conditions with modulation of visual information and mental attention and discuss the resulting outcome while comparing methods. Seventeen healthy individuals were asked to keep the head steady in space while subjected to pseudorandom rotational perturbations in the horizontal plane, firmly seated on an actuated chair. Both methods confirmed the results for gain in previous studies showing fair ability to keep the head steady in space below 1 Hz with vision. Compensation deteriorated when vision was removed and worsened further with addition of a mental task. Between 1 and 2 Hz, unity gain occurred between head and trunk movements, whereas above 2 Hz the head moved more than the trunk. For phase angles, the original method demonstrated a phase split occurring from ~1 Hz, a purely mathematical artifact that caused subjects with virtually identical movements to appear as significantly different. This artifact was eliminated by analyzing the head‐room relative to trunk‐room rather than head–trunk relative to trunk‐room angles, thus preventing potentially erroneous interpretations of the results.

## Introduction

The ability to keep the head steady in space when the body is moving serves a purpose in order to keep the visual field stable on the retina (Ortega et al. 2009). In animals such as owls (Money and Correia 1972) and pigeons (Gioanni 1988; Mittelstaedt 1997), where eye movement is minimal, the reflexes controlling the head are robust, whereas the role of reflex control of the head has been described as less significant in humans (Guitton et al. 1986) probably because of considerably greater oculomotor range (Smith et al. 2014). In humans the stability of the visual field on the retina is provided by the vestibulo‐ocular, cervico‐ocular, and optokinetic reflexes (Kelders et al. 2003). Nevertheless, the ability to keep the head steady in space as the frame of reference containing the visual and vestibular systems is essential for control of movement also in humans (Pozzo et al. 1990), and coordinated head movements are essential for control of gaze shifts and gaze stabilization (Borel et al. 1994).

Two reflex systems are proposed to contribute to stability of the head and neck plant by regulating neck muscle stiffness; (1) the vestibulocollic reflex (VCR) keeps the head stable in space by vestibular neurons projecting to neck motor neurons (Wilson and Schor 1999), and (2) the cervicocollic reflex (CCR) keeps the head and neck stable in relation to the trunk by means of proprioceptive input from muscle spindles (Peterson 2004). These two reflex systems may work in a reciprocal manner in order to stabilize the head position during perturbations either from intentional movements such as walking, or perturbations from external sources. The VCR produces compensatory head movements in the opposite direction to the perturbation by activating the neck muscle forces against the direction of perturbation and inhibiting muscle force working in the same direction as the perturbation. In contrast, the CCR activates the muscles working with the direction of the perturbation when those are exposed to stretch. During voluntary movements, the reflexes would have to be cancelled for the head to move freely (Roy and Cullen 2001; 2004), alternatively reflex excitability may be modulated by voluntary activity and serve to dampen the oscillations created by the mass‐spring system of the head and neck (Peng et al. 1996). In order to keep the head steady in space rather than on the trunk during perturbations to the body, the head has to counter rotate relative to the trunk with the same amplitude. Although reflex control of the head has been investigated in both animal models and humans, as well as modeled by simulations, the theoretical assumptions about the function still remain to be directly demonstrated in experimental settings (Goldberg and Cullen 2011).

A model to study the approximate contributions of voluntary and reflex mechanisms for control of the head and neck in humans was proposed by Keshner and coworkers in 1995. Head stability was examined in healthy subjects exposed to pseudorandom rotations in the horizontal and in the sagittal plane, respectively, while seated with the trunk in a fixed position and the head allowed to move freely. The authors of those studies used the term “compensation” to describe the effort to maintain head stability in space, that is, suppression of the disturbance from the rotating chair; we will use the same convention in the present paper. During rotations in the horizontal plane, with as well as without a visual reference point, good or fair compensation has been found up to 1 Hz (Keshner and Peterson 1995). A drop in gain between 1 and 2 Hz was interpreted by the authors as “interference” between control systems. In a dual condition with a concomitant arithmetic task and no vision, subjects showed poor compensation below 1 Hz, but increasing compensation was apparently identified between 1 and 2 Hz (Keshner and Peterson 1995; Keshner et al. 1995). At frequencies below 1 Hz, reflex mechanisms were considered negligible (Keshner and Peterson 1995; Keshner et al. 1995), which had also been previously shown by Guitton and colleagues in a similar protocol (Guitton et al. 1986).

At frequencies above 3 Hz, experimental studies as well as simulations have shown that mechanical resonance occurs and that neither voluntary nor reflex systems are able to control the head position when exposed to unpredictable perturbations at such high frequencies (Keshner and Peterson 1995; Keshner et al. 1995; Peng et al. 1996, 1999).

Previous studies on head stability in space (Guitton et al. 1986; Keshner and Peterson 1995; Keshner et al. 1995) have analyzed the angular position or velocity of the head with respect to the trunk. This study aimed at repeating the experimental protocol described by Keshner and Peterson (1995), who analyzed the angle of the head with respect to the trunk. However, when processing larger datasets, unexpected artifacts emerged and it was established that this method produces ambiguous results with potential consequences for physiological interpretations. This problem may be solved by using the room, rather than the trunk, as a frame of reference for the head angle. Using the room as the frame of reference was also suggested by (Goldberg and Cullen 2011). The aim of the study was to investigate the approximate contribution of reflex and voluntary control across perturbing frequencies and conditions with modulation of visual information and mental attention and discuss the resulting outcome while comparing methods for analysis.

## Methods

### Participants

Seventeen healthy subjects [31.5 (7.7) years; height 1.74 (0.1) m; weight 73.0 (14.1) kg, 9 males] without any neck and shoulder complaints participated in the study. Exclusion criteria were reduced vision or uncorrected vision disorders, diagnosed musculoskeletal or neurological condition. The study was approved by the Regional Ethics Committee (2011/2522/REK) and conducted in agreement with the Helsinki declaration. All participants signed an informed consent before entering the study.

### Data acquisition

The rationale for the choice of stimuli was to target presumed voluntary as well as reflex‐based systems for control of head stability across a range of frequencies under different conditions. The task at hand was to keep the head steady in space while the body was exposed to pseudorandom rotations in the horizontal plane. Each subject was exposed to one trial (duration 200 s) of each of three conditions in the following order; with vision (VS), without vision (NV), and without vision with an additional mental task counting backwards from 500 in steps of seven (MA), the latter in order to keep divert the attention from control of head position. In the NV and MA conditions the subject was blindfolded. The first condition (VS) aimed to investigate voluntary control with an available visual reference to the current position. This reference was provided by a laser pointer mounted in a rigid fixture on top of the head of the participant, which was aimed toward a vertical line on a white surface (distance 1.6 m) in front of the subject. A 5 cm intersecting horizontal line guided the projected laser beam in order to keep the head stable in neutral position and the laser beam aligned with the horizontal plane. It is difficult to accurately maintain a head‐based laser on an earth‐fixed target; thus this condition implies considerable subject effort to control the laser position. The second condition (NV) challenged voluntary control without visual information. The purpose of the third condition (MA) was to investigate the contribution of reflex control.

**Figure 1:**
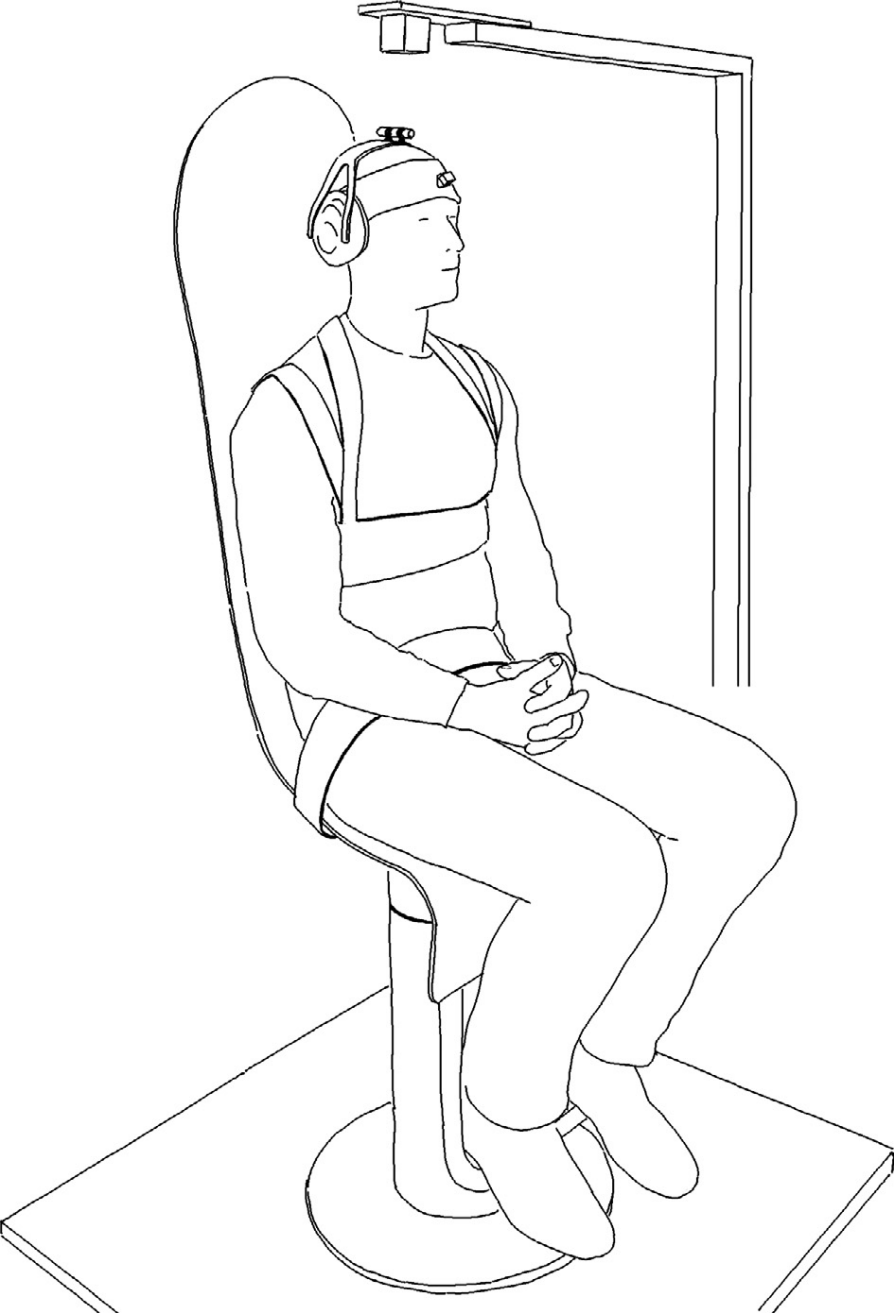
An instrumented subject strapped to the actuated chair. The cube above the subject's head represents the electromagnetic transmitter. Note that the ear muffs have holes leaving the ears uncovered to enable communication with the subject and not induce further modulation of sensory input with potentially unknown effect.

Sinusoidal rotations around the vertical normal axis were transferred to the trunk by means of an actuated chair (see Figure 1 and supplementary material). The rotational axis of the chair coincided approximately with the axis of the cervical spine. The participant was seated in a vertical upright position on the chair with feet on a foot rest and trunk and legs firmly strapped to the high back rest and seat of the chair to minimize movement between the body and the chair. Cross correlations from pilot studies assured that the response of the trunk corresponded to that transferred by the chair (*φ*_xy_ (τ* *= 0) = 0.95).

Following the protocol from Keshner and Peterson (1995), the perturbation signal was a sum‐of‐sines pattern, presently with ten harmonic components. The fundamental frequency was set to *F* = 0.005 Hz and the harmonics were chosen as the prime numbers

H=37,49,71,101,143,211,295,419,589,823

The sinusoids of relative primes provided pseudorandom perturbations in a pattern without repetitions over the fundamental period of *T *= 200 s. Such a pattern was chosen to avoid anticipatory preparation in the subjects and harmonic contamination between the resulting frequencies, which ranged from 0.185 to 4.117 Hz. Chair velocity amplitudes were decreased as frequency increased: 20^o^/s from 0.185 to 0.355 Hz, 19^o^/s from 0.505 to 1.055 Hz, 16^o^/s from 1.475 to 2.095 Hz, 15^o^/s at 2.945 Hz, and 13^o^/s at 4.115 Hz. The lowest frequency (0.185 Hz) corresponds to the largest harmonic angular excursion of ± 17^o^ (the maximum theoretical excursion being somewhat larger due to superposition of all harmonics). The same waveform was used for all conditions and all participants. The sum‐of‐sines angular velocity excitation signal, denoted by *u*(*t*), may be described by the function

$$u\left(t\right)=\sum_{k\in H}a_{k}\sin\left(2\pi Fkt+\phi_{k}\right),$$

where *k* represents each of the harmonics, *a*_*k*_ is the amplitude of the *k*’th harmonic (in radians/second), *t* is time (in seconds), and *φ*_*k*_ is the phase angle (in radians) of the *k*’th harmonic at *t* = 0 In the current case *Φ* = 0∀*k*, but the actual values used are believed to be irrelevant for the results and therefore *φ*_*k*_ is left out of the equations in the following.

This sinusoidal rotations transferred from the actuated chair to the trunk and head, is given approximately by the formulae

$$\theta^{T}\left(t\right)\approx \sum_{k\in H}A_{k}^{T}\sin\left(2\pi Fkt\right)$$

$$\theta^{H}\left(t\right)\approx \sum_{k\in H}A_{k}^{H}\sin\left(2\pi Fkt\right)$$

The superscripts here signify whether it is the trunk (*T*) or head (*H*) angle that is in question. To signify that these angles are both measured with respect to a room‐fixed coordinate frame, the quantities *θ* ^*T*^ and *θ* ^*H*^ will be referred to as the trunk‐room angle and the head‐room angle, respectively. Similarly, the angle of the head with respect to the trunk will be referred to as the head–trunk angle. Note that these measured signals will include frequency content (noise) not present in the excitation signal, implying that the above formulations are not exact, hence the approximation signs.

Also note that the excitation signal is defined in terms of the angular velocity *u*(*t*) while θ^T^ (*t*) and *θ* ^*H*^ (*t*) are angles. The above formulae still holds, as the angular excursion amplitude coefficients (*A*_*k*_)relate to the angular velocity amplitude coefficients as $A_{k}=-\frac{1}{2\pi Fk}a_{k},k\in H$ .

Data for rotations in the horizontal plane of the chair, trunk, and head were collected at 240 Hz by a Liberty electromagnetic motion tracking system (Polhemus, Colchester, VT). A transmitter was placed ~ 20 cm above the head of the subject. Rotations of the chair were registered by a sensor fixed to the back rest. The sensor monitoring the rotations of the trunk was placed approximately at the level of the 2nd thoracic vertebrae by means of an adjustable vest and the sensor monitoring the head was attached to the forehead by a sturdy elastic headband (Figure 1). The rotation was driven by a brushed DC motor with a 1:308 gear ratio (Maxon Motor, Sachseln, Switzerland, part no. 353295), controlled by a LabVIEW program via a NI 9505 DC Brushed Servo Drive (both of National Instruments Corporation, Austin, TX). The main structure of the chair, including casings and main bearing, was built from nonmetallic materials in order to minimize their influence on the electromagnetic motion capture system used. The DC motor and gear were placed close to the floor and power electronics was placed at a 2 m horizontal distance from the base of the chair for the same reason.

### Data analysis

**Figure 2:**
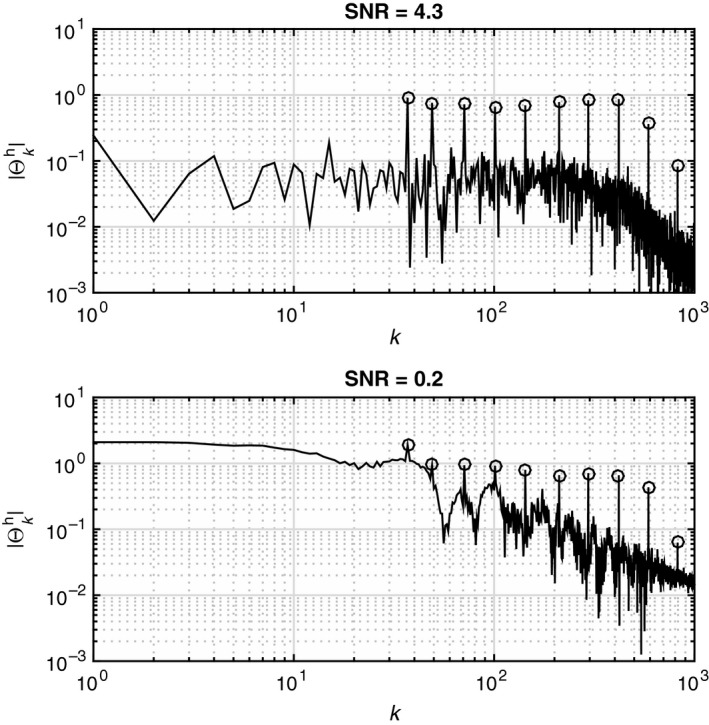
Spectral magnitudes of head movements in response to rotational perturbations in the horizontal plane showing signal‐to‐noise ratio (SNR). Excitation harmonics are indicated by circles. Note that the noise floor is predominantly flat, resembling white noise without any notable overharmonics of the excitation frequencies. This justifies treatment of the system as linear, as any significant nonlinear effects would have caused “harmonic leakage” in the output signals.

**Figure 3:**
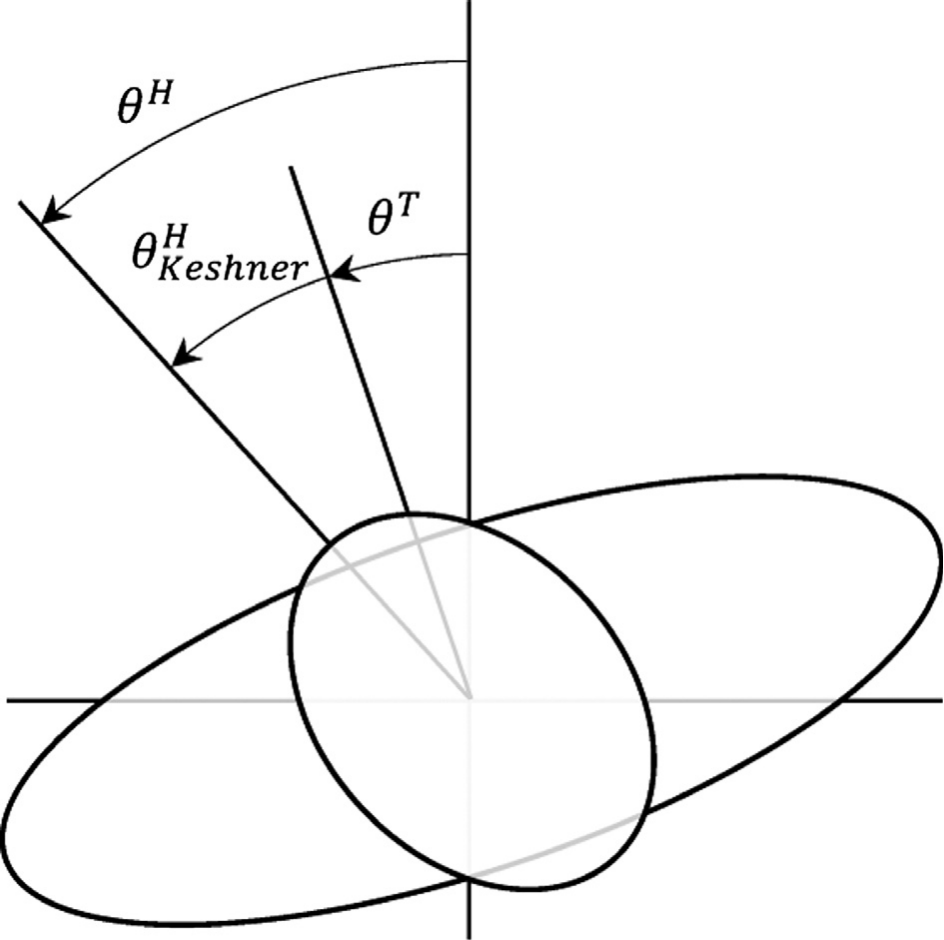
Illustration of the differences between the mathematical models; Keshner and Peterson (1995), where head movements were measured relative to the trunk ($\Theta_{\mathrm{Keshner}}^{H}$ ), and the model used in this study where the head angle was measured relative to the room (*θ* ^*H*^).

Information about the subjects’ motor responses to the perturbation was extracted with spectral analysis. In this study, the Matlab function “*P = angle(Z)”* was used which returns the phase angles, in radians, for each element of complex array *Z*. The angles lie between ±*π*. Under the assumption of linearity, the motion of the head and trunk would be a sum‐of‐sines in the excitation frequencies. This assumption was validated with analyses of the spectral magnitudes for the head‐room and trunk‐room angles, showing satisfactory signal‐to‐noise ratio (SNR), albeit with quite notable noise levels for the head‐room angle (Figure 2). The excitation response of a linear dynamic system may be modeled as a complex transfer function, which describes the frequency response of a system, that is, the gain and phase shift of the output (response) signal relative to the input (excitation) signal. Such a function may be expressed as

$$G\left(j2\pi f\right)\frac{Y(j2\pi f)}{U(j2\pi f)}\left(\frac{\text{Response}}{\text{Excitation}}\right),$$

where *Y* and *U* are polynomial functions, *j* is the imaginary unit, and *f* is frequency in Hz. The transfer function relating the head‐room angle (response) to the trunk‐room angle (excitation) was estimated by calculating a Fourier series of the recorded time series over the excitation frequencies. Thus, in this study, the compensatory neck movements were related to the room, not to the trunk (Figure 3) as described in previous studies (Guitton et al. 1986; Keshner and Peterson 1995; Keshner et al. 1995). This is a crucial choice because in the latter case, arbitrary and tiny variations in the dataset may cause significant changes in the calculated gain and phase due to a mathematical peculiarity, as described further in the Discussion. For a description of the analysis using the trunk as the frame of reference for the movement of head, see Keshner and Peterson 1995. Below follows a description of the analysis using the room as the frame of reference.

Computation of the following integrals furnished a complex signal description at the *k*’th harmonic:

$$\Theta_{k}^{T}=\frac{2}{T}\int_{0}^{T}\theta^{T}\left(t\right)e^{-j2\pi Fkt}dt,\Theta_{k}^{H}=\frac{2}{T}\int_{0}^{T}\theta^{H}\left(t\right)e^{-j2\pi Fkt}dt$$

The transfer function was subsequently evaluated at the discrete excitation frequencies by evaluating

$$G_{k}=\Theta_{k}^{H}/\Theta_{k}^{T},k\in H$$

Gain and phase shifts of the head‐room angle relative to the trunk‐room angle were recovered by taking the absolute value and argument (angle) of this complex transfer function, as follows:

$$\frac{A_{k}^{H}}{A_{k}^{T}}=\left|G_{k}\right|,\phi_{k}^{H}-\phi_{k}^{T}=\text{arg}\;\;G_{k}$$

Resulting transfer functions are presented as Bode plots with gain and phase shown for the 10 excitation frequencies. The Bode plot in an ideal way decouples the system properties of gain, phase shift, and time constants/eigenfrequencies, which allows direct comparison and statistical analyses of linear systems with different dynamics. Thus, our statistical analysis is also based on Bode data (i.e., logarithmic gain and linear phase).

Perfect compensation for the head in response to the perturbations is represented by a gain of zero, that is, the head is kept stationary in space and thus has no angular amplitude relative to the room at the excitation frequency in question. This is achieved by head‐trunk rotations of the same amplitude as, but in the opposite direction of, that of the trunk‐room angle. Note that with regard to the dynamic properties of compensatory head movements generated by voluntary, reflex, and mechanical mechanisms, total compensation, that is, zero gain and 0^o^ phase angle is not practically attainable. The voluntary system is likely to produce a compensatory signal with phase opposite to that of the imposed rotation of the trunk with a delay of approximately 0.2 s. (Keshner and Peterson 1995). A gain of unity (=1) indicates that the head moves in space with the same amplitude as the trunk, and gain >1 denotes that the head moves more than the trunk relative to space. Perfect temporal compensation in response to perturbations is shown as 0^o^ shift for phase angles; positive values denote phase lead and negative values show phase lag. The transfer functions that were identified and analyzed in the present work encode the linear dynamic relation between trunk motion (input) and head motion (output). Once in possession of such transfer functions, motion synthesis is also possible. An arbitrary input signal may be furnished and the transfer function can be used to predict the corresponding output signal. A video in the supplementary files shows the case of sinusoidal input signals in ascending frequency. A representative transfer function obtained from a single participant has been used. A transfer function from chair to trunk motion was used to animate the blue oval, whereas the transfer function from chair to head motion is animated by the oval with red perimeter. The chair motion used as input is shown by the gray rectangle. A black line serves as a fixed room reference. The input signals have been exaggerated so as to produce discernible head motion. They do not correspond to the actual magnitude of chair motion used in the experiment.

### Statistical analysis

Kinematic rotational data generally need to be treated with special statistical methods, for example, the Cosine statistics (Stavdahl et al. 2005), that account for the inherent cyclicity of rotations. However, for comparison of phase shifts of different transfer functions, traditional statistical methods were employed in order to avoid, for example, treating two phase angles as the same if they differ by a multiple of 2π radians. The statistics were generated with SPSS 22.0 (Statistical Package for the Social Sciences, Inc., Chicago). Data were inspected with histograms and normal distribution was confirmed with P–P and Q–Q plots. For each method, separate general linear models for gain and phase were set up for repeated measures with conditions as within subject factors (*n* = 3, VS, NV, MA) with the harmonics as measures within each factor (*n* = 10: frequencies 0.185‐4.115 Hz). Multivariate analyses (Wilks’ Lambda) were used for within subject's comparisons across conditions and frequencies. Bonferroni corrected contrasts were used for pairwise comparisons between conditions for each frequency. Correction for sphericity was applied when called for (Huynh–Feldt). The alpha‐level of significance was set at *P < *0.05.

## Results

**Figure 4:**
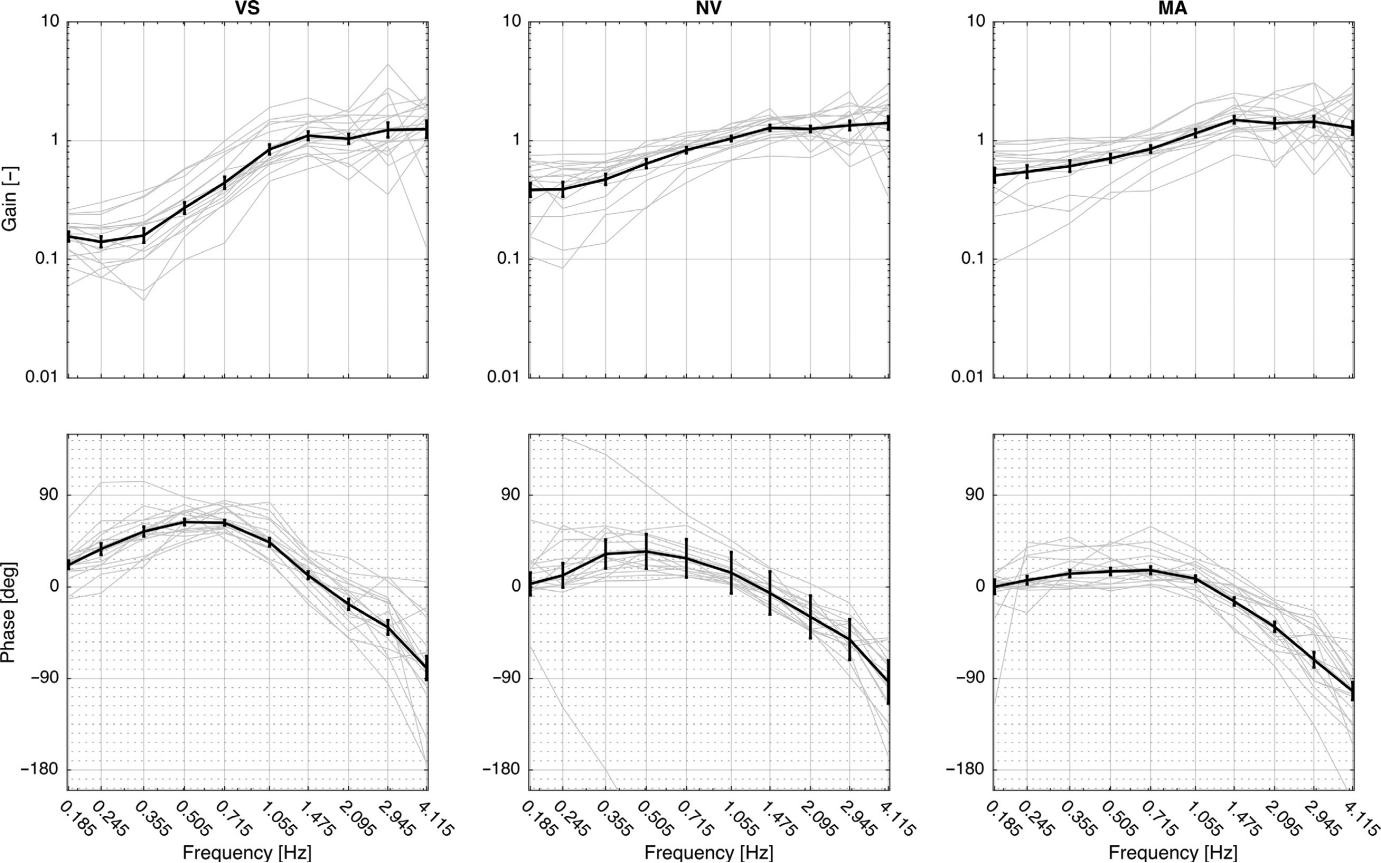
Bode diagrams of transfer functions according to the analysis method advocated in this study (head‐room relative to trunk‐room) for the three conditions with vision (VS), without vision (NV), and without vision with a cognitive task (MA), respectively. Mean and SEM. Gray curves in the background show the individual responses.

**Figure 5:**
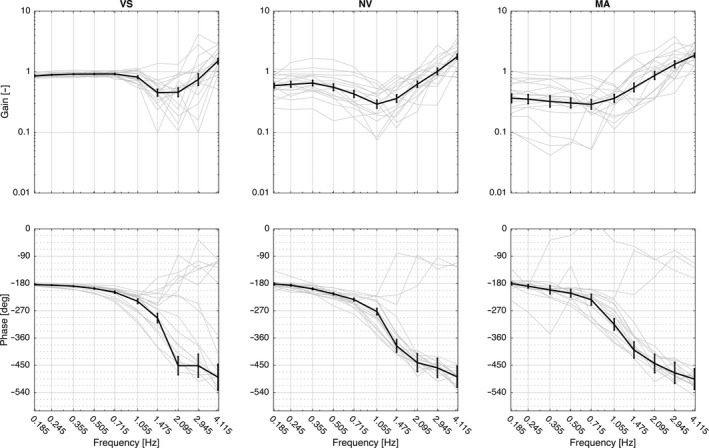
Bode diagrams of transfer functions according to Keshner and Peterson (1995) (head‐trunk relative to trunk‐room) for the three conditions with vision (VS), without vision (NV), and without vision with a cognitive task (MA), respectively. Mean and SEM. Gray curves in the background show the individual responses. Note the increasing magnitude of SEM and the phase spilt at higher frequencies.

Figures 4 and 5 illustrate the curves for gain and phase for the two methods; the movement of the head relative to the room as the frame of reference and to the trunk as the frame of reference, respectively. Tables 1 and 2 show the *F*‐statistics for comparison between methods for within subjects’ effects of condition across frequencies. Estimates are not included, as numerical results are not comparable between methods.

**Table 1:** Within subjects’ effects of condition across frequencies for gain compared between methods and displayed with *F*‐statistics as estimates are not comparable between methods. *Trunk* refers to the original method where the trunk is the frame of reference for the head. *Room* refers to the alternative method where the room is the frame of reference for the head

Frequency	VS versus NV		NV versus MA	
	Trunk	Room	Trunk	Room
0.185	***F*** ** = 21.6, ** ***P *** **<** *** *** **0.001**	***F*** ** = 79.8, ** ***P *** **<** *** *** **0.001**	***F *** **=** *** *** **12.2, p = 0.003**	***F *** **=** *** *** **6.7, ** ***P *** **=** *** *** **0.020**
0.245	***F *** **= 14.4, ** ***P *** **= 0.001**	***F*** ** = 47.4, ** ***P *** **<** *** *** **0.001**	***F *** **=** *** *** **12.7, ** ***P *** **=** *** *** **0.002**	***F *** **=** *** *** **6.3, ** ***P *** **=** *** *** **0.023**
0.355	***F*** ** = 12.7, ** ***P*** ** = 0.002**	***F*** ** = 57.8, ** ***P *** **<** *** *** **0.001**	***F *** **=** *** *** **13.0, ** ***P *** **=** *** *** **0.002**	*F *=* *3.6, *P *=* *0.077
0.050	***F*** ** = 16.5, ** ***P*** ** = 0.001**	***F*** ** = 46.0, ** ***P *** **<** *** *** **0.001**	***F *** **=** *** *** **12.3, ** ***P *** **=** *** *** **0.003**	*F *=* *0.9, *P *=* *0.351
0.175	***F*** ** = 37.9, ** ***P *** **<** *** *** **0.001**	***F*** ** = 25.2, ** ***P *** **<** *** *** **0.001**	***F *** **=** *** *** **6.6, ** ***P *** **=** *** *** **0.020**	*F *=* *0.1, *P *=* *0.808
1.055	***F*** ** = 47.7, ** ***P *** **<** *** *** **0.001**	***F*** ** = 4.8, ** ***P*** ** = 0.044**	*F *=* *2.1, *P *=* *0.168	*F *=* *2.6, *P *=* *0.128
1.475	*F* = 1.3, *P* = 0.267	*F* = 2.3, *P* = 0.149	***F *** **=** *** *** **9.0, ** ***P *** **=** *** *** **0.008**	***F *** **=** *** *** **7.3, ** ***P *** **=** *** *** **0.016**
2.095	***F *** **= 4.6, ** ***P *** **= 0.046**	*F *=* *4.3, *P* = 0.055	***F *** **=** *** *** **8.9, ** ***P *** **=** *** *** **0.008**	*F *=* *2.9, *P *=* *0.105
2.945	*F* = 3.0, *P* = 0.099	*F *=* *0.0, *P* = 0.350	***F *** **=** *** *** **10.9, ** ***P *** **=** *** *** **0.004**	*F *=* *1.7, *P *=* *0.205
4.115	***F*** ** = 8.6, ** ***P*** ** = 0.009**	*F *=* *1,5, *P* = 0.237	*F *=* *1.3, *P *=* *0.265	*F *=* *1.3, *P *=* *0.274

VS, with vision; NV, without vision; MA, without vision and additional mathematical task. *P*‐value in bold denote a significant difference between tasks.

**Table 2:** Within subjects’ effects of condition across frequencies for phase compared between methods and displayed with *F*‐statistics as estimates are not comparable between methods. *Trunk* refers to the original method where the trunk is the frame of reference for the head. *Room* refers to the alternative method where the room is the frame of reference for the head

Frequency	VS versus NV		NV versus MA	
	Trunk	Room	Trunk	Room
0.185	*F *=* *0.4, *P *=* *0.055	*F *=* *0.1, *P *=* *0.742	*F *=* *0.0, *P *=* *0.900	*F *=* *1.0, *P *=* *0.175
0.245	*F *=* *0.8, *P *=* *0.375	*F *=* *3.4, *P *=* *0.082	*F *=* *0.9, *P *=* *0.366	*F *=* *0.8, *P *=* *0.388
0.355	***F *** **=** *** *** **27.0, ** ***P *** **<** *** *** **0.001**	***F *** **=** *** *** **5.4, ** ***P *** **=** *** *** **0.034**	*F *=* *0.0, *P *=* *0.909	*F *=* *0.3, *P *=* *0.612
0.050	***F *** **=** *** *** **64.5, ** ***P *** **<** *** *** **0.001**	***F *** **=** *** *** **6.6, ** ***P *** **=** *** *** **0.021**	*F *=* *1.5, *P *=* *0.236	*F *=* *0.0, *P *=* *0.995
0.175	***F *** **=** *** *** **46.0, ** ***P *** **<** *** *** **0.001**	***F *** **=** *** *** **8.1, ** ***P *** **=** *** *** **0.012**	*F *=* *0.4, *P *=* *0.534	*F *=* *0.2, *P *=* *0.630
1.055	***F *** **=** *** *** **20.0, ** ***P *** **<** *** *** **0.001**	***F *** **=** *** *** **5.4, ** ***P *** **=** *** *** **0.034**	*F *=* *0.0, *P *=* *0.853	*F *=* *0.6, *P *=* *0.460
1.475	***F *** **=** *** *** **7.2, ** ***P *** **=** *** *** **0.016**	*F *=* *3.1, *P *=* *0.100	*F *=* *0.0, *P *=* *0.819	*F *=* *0.4, *P *=* *0.518
2.095	***F *** **=** *** *** **6.1, ** ***P *** **=** *** *** **0.025**	*F *=* *2.1, *P *=* *0.169	*F *=* *0.0, *P *=* *0.953	*F *=* *0.3, *P *=* *.0615
2.945	*F *=* *3.9, *P *=* *0.065	*F *=* *2.5, *P *=* *0.131	*F *=* *0.0, *P *=* *0.820	*F *=* *0.0, *P *=* *0.866
4.115	*F *=* *2.8, *P *=* *0.112	*F *=* *1.8, *P *=* *0.196	*F *=* *0.0, *P *=* *0.821	*F *=* *0.0, *P *=* *0.848

VS, with vision; NV, without vision; MA: without vision and additional mathematical task. *P*‐value in bold denote a significant difference between tasks.

### Gain with the room as the frame of reference

Gain differed significantly between conditions across frequencies (*F*_10,46_ = 8.1, *P *<* *0.001, Figure 4). In VS gain was close to 0.1 at 0.185 Hz and stayed <1 at frequencies below 1 Hz, implying that the head was kept relatively stationary in space in this frequency range. Between 1.475 and 2.095 Hz the mean gain was ~1, denoting that the head moved with the same amplitude as the trunk. At frequencies above 2.095, mean gain exceeded 1 and the head moved more than the trunk relative to space. In NV gain stayed < 1 below 1 Hz but was significantly closer to 1 than in VS (Table 1). At 1.055 Hz, the mean gain was ~1 and at higher frequencies exceeding 1. There were no significant differences between VS and NV between 1.055 and 4.115 Hz. In MA gain was getting closer to 1 and was significantly higher compared with NV for the two lowest frequencies. Above 0.715 Hz gain was exceeding 1 and generally similar to NV, albeit significantly lower than NV at 1.475 Hz

### Gain with the trunk as the frame of reference

Gain differed significantly between conditions across frequencies (*F*_10,46_ = 5.2, *P *<* *0.001, Figure 5). In VS gain was close to 1 between 0.185 and 1.055 Hz implying that the head was kept relatively stationary in space in this frequency range. Between 1.055 and 1.475 Hz gain was reduced indicating that the head moved less relative to the trunk, and thus more relative to the room. Gain increased after 2.095 Hz and the head moved more than the trunk after 2.945 Hz. In NV, gain was lower than in VS up to 1 Hz, increased at 1.055 Hz and was higher than in VS at 2.095 and 4.115 Hz (Table 1). There were no significant differences between VS and NV between 1.475 and 2.945 Hz. In MA, gain was lower compared with NV between 0.185 and 0.175 Hz, increased above 0.715 Hz and was in general significantly higher than in NV between 1.475 and 2.945 Hz, except at 1.055 and 4.115 Hz.

### Phase with the room as reference

Phase differed significantly between conditions across frequencies (*F*_20,46_ = 7.0, *P *<* *0.001, Figure 4). In VS there was a clear increase in phase lead from 0.185 Hz to 0.505 Hz, denoting that the head moved somewhat ahead of the trunk relative to the room. Above 0.715 Hz the lead was decreasing and phase was close to 0^o^ between 1.475 and 2.095 Hz, indicating that the head moved approximately in phase with the trunk. Above 2.095 Hz there was an increasing phase lag as the movement of the head was increasingly lagging behind the movement of the trunk. In NV there was a steady phase lead up to 0.505 Hz. Between 0.715 and 1.055 the phase was close to 0^o^. At frequencies above 1.055 there was an increasing phase lag. There was a significantly greater phase lead in VS compared to NV between 0.355 and 1.055 (Table 2). The mean phase curve for the MA condition followed the general trend of the NV condition, without any statistical difference between the two.

### Phase with the trunk as reference

Phase differed significantly between conditions across frequencies (*F*_10,24_ = 7.0, *P *<* *0.001, Figure 5). An increasing phase lag was seen with increasing frequency. There was no difference between VS and NV for the two lowest and the two highest frequencies (Table 2). Between 0.335 and 2.095 Hz, the phase lag was greater in NV than in VS. There were no significant differences between NV and MA.

## Discussion

In this study, we have repeated the experimental protocol for head steadiness in space during horizontal perturbations to the trunk as described by Keshner and Peterson (1995). For comparison between our method presented in this study (head‐room relative trunk‐room) and the original analysis described by Keshner and Peterson (1995) (head‐trunk relative trunk‐room) (Figure 3), the same dataset was treated according to both methods and statistical analyses performed for within subject's effects of condition in each separate method. The analysis according to our method presented in this study produced curves for gain that were quite different compared to the curves produced with the original method as well as to the results in Keshner and Peterson (1995). This is explained by the different frames of reference used in the two methods. For illustration, consider the case of perfect compensation, that is, the head is kept stationary in space. When the trunk is used as the frame of reference for the head movement, the amplitudes of the head and trunk will now be identical (1/1 = 1, Figure 5).With our method when the room is used as the frame of reference for the head movement, this results in a gain of zero (Figure 4). In spite of the differing numerical and graphical results produced by the different calculations, statistics for within subject's comparisons shows similar effects of condition for each one of the two methods. For gain at frequencies below 1 Hz, results produced with calculations according to the two different methods were in agreement corroborated by findings of Keshner and Peterson (1995), as well as Guitton et al. (1986). Gain significantly lower than one using our method and close to one according to the original method implies that the head was held relatively stationary in space, indicating fair compensation. Without vision, NV, compensation was reduced, moving away from zero gain with our method, whereas reduced compensation was marked by a falling gain using the original method. In the MA condition without vision and with an additional cognitive task, our method showed that gain was close to 1 and phase close to 0, indicating that the head moved approximately with the trunk. With the original method, approximately the same results are indicated by reduced gain and phase close to ‐180.

At frequencies above 1 Hz, the head moved with the same amplitude as the trunk shown as gain approaching one in Figure 4 and as a fall in gain in Figure 5. This occurred in all three conditions, albeit tending to start at lower frequencies in NV and MA. At frequencies above 2 Hz, both methods indicated gain exceeding unity (i.e., the head moved more than the trunk), which was in line with the results of Keshner and Peterson (1995).

In agreement with previous authors we interpret the results using our method in terms of voluntary control prevailing at frequencies below 1 Hz (Guitton et al. 1986; Keshner and Peterson 1995). It has been suggested that voluntary control overrides reflex control in order not to oppose intended motion (Roy and Cullen 2001). Results from the VS and NV conditions do, however, not exclude the influence of reflex control in order to reduce oscillations and improve dynamic stability during voluntary movements by increasing muscle stiffness (Peng et al. 1996). When diverting the attention by a cognitive task and removing vision, such as in MA, gain was getting closer to 1 and phase closer to 0, indicating that the head was moving more with the trunk at all frequencies. This can be interpreted as a reflex contribution to head‐on‐trunk stabilization when the trunk is perturbed even at relatively low frequencies, suggesting that the VCR and CCR may be working in a reciprocal manner resulting in cocontraction between agonists and antagonists (Wilson and Schor 1999; Peterson 2004). This notion is further corroborated in a study of Keshner and Peterson (1995), by electromyography of the semispinalis, splenius capitis, and sternocleidomastoid muscles, showing constant activity during the MA condition at 0.1 Hz. For frequencies >2 Hz, our method showed that the head moved more than the trunk with increasing phase lag, indicating that a resonance effect was emerging and that neither system was able to stabilize head position. This is in agreement with other studies (Keshner and Peterson 1995; Keshner et al. 1995; Peng et al. 1996, 1999).

The most obvious difference between our and the original method of analyses occurred for phase. With our method, a phase lead was demonstrated for all three conditions at frequencies up to 1 Hz, and turned toward an increasing phase lag as frequencies increased further. In contrast, using the original method showed an increasing phase lag for the majority of the cases and a clear phase split at 0.715 Hz (Figure 5). Keshner and Peterson (1995) demonstrated good compensation for VS and NV up to 0.715 Hz (see Figure 3 in Keshner and Peterson 1995), then a phase drop and thereafter from 1.475 Hz, a distinctive 90° positive phase shift from −180° to −90° shown as a steep downward shift of the curve followed by a steep incline.

**Figure 6:**
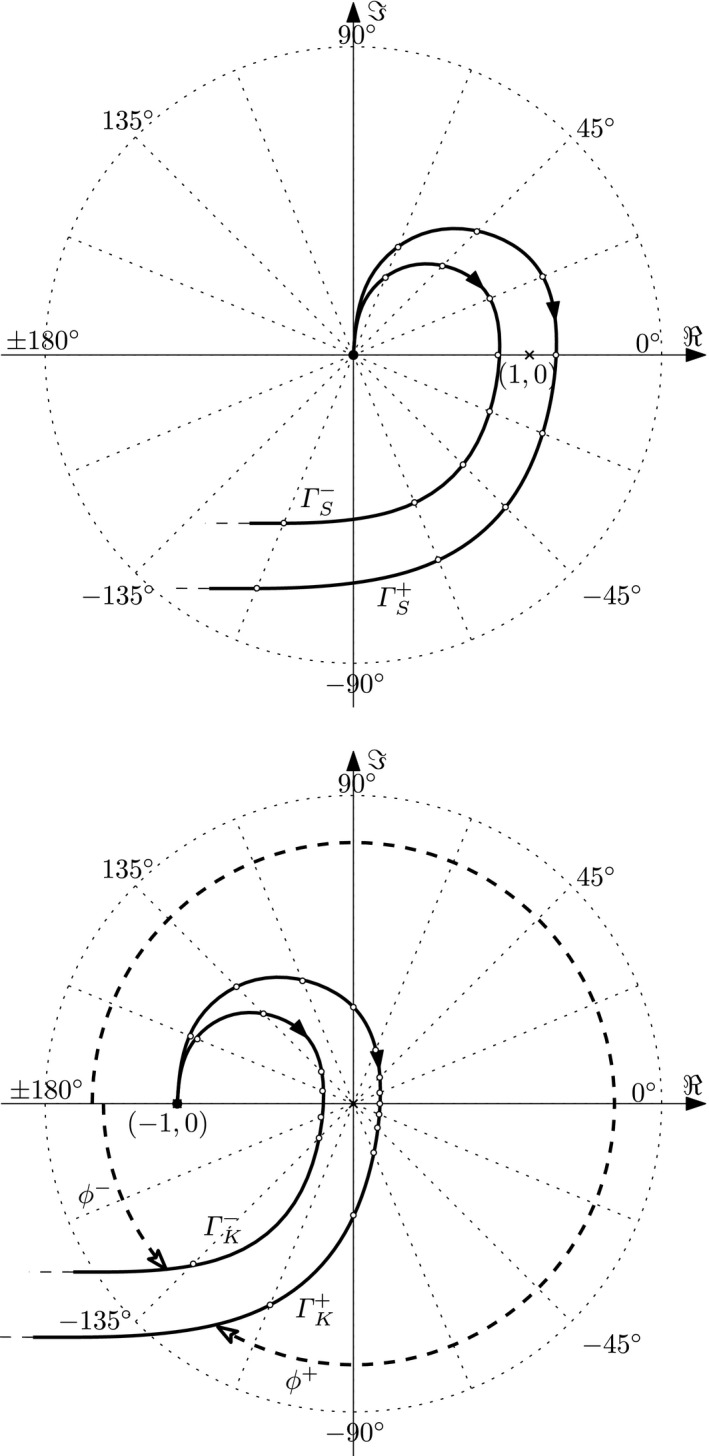
Graphical representations in the complex plane of the transfer functions of the two different methods of analysis based on an arbitrary dataset; the present method measuring the head‐room relative to the trunk‐room (upper graph) and the method presented by Keshner and Peterson (bottom graph). See the main text for interpretation.

Thus, using the curves resulting from defining the trunk as the frame of reference for movements of the head (Figure 5) may be interpreted in terms of some subjects exhibiting behavior significantly different from others. In the study by Keshner and Peterson (1995) only seven subjects participated and some outliers can be seen, in particular for phase in the MA condition. However, the number of subjects in that study was not sufficient to fully show the adverse effect of the method. As there were more than twice as many subjects in this study, the effect of analyzing the head relative to the trunk becomes obvious, as illustrated in the bottom part of Figure 6. Due to minor individual differences and measurement noise, some estimated transfer functions will exhibit gain values slightly larger than the mean while others will attain somewhat smaller gain values. These two cases are represented by the graphs denoted Γ_*K*_^+^ and Γ _*K*_^−^, respectively. Now consider the angles of two vectors from the point of origin to a point on each of the curves as these points move in the direction of the arrowheads. When the two curves pass the origin on opposite sides, the angle of the Γ _*K*_^−^ vector moves in the opposite direction to that of Γ_*K*_^+^, creating an artificial phase difference of 360° that explains the phase split demonstrated in Figure 5. By analyzing the position of the head relative to space by utilizing the head‐room relative trunk‐room approach, this has the effect of adding +1 to the equation. This corrects the starting point of the dataset to the origin of the complex plane (upper part of figure 6). In this case the two resulting graphs, denoted Γ_*S*_^+^ and Γ _*S*_^−^, respectively, are qualitatively similar in that they pass the origin on the same side, thus removing the mathematical artifact causing the phase split. As a consequence, variability is also reduced both for gain and phase and the apparently significant (but in fact insignificant) difference between subjects is removed.

Thus, the two methods applied to the very same dataset will result in different curves for gain as well as for phase. Although strictly mathematically one is no more correct than the other, the physiological interpretation may be erroneous when applying the analyses of the head‐trunk angle. This becomes particularly obvious between 1 and 2 Hz, where Keshner and Peterson (1995) show a drop in gain and an advance in phase, indicating reflex control stabilizing the head on the trunk. When using their model for calculation with the trunk as the frame of reference on our dataset with more than twice as many participants, variability became very large for gain as well as phase. A split in positive and negative directions emerged for phase where most subjects displayed a phase lag (Figure 5). This obscures whether the head follows the trunk or not and consequently may lead to ambiguous interpretations of the results. Furthermore, the phase split also (falsely) indicate opposite behavior in groups of subjects. When applying the present method using the room as the frame of reference, the phase split disappeared and variability for phase as well as gain was dramatically reduced; responses between 1 and 2 Hz gain was close to one and the phase curve intersected with zero indicating that the head followed the trunk. This corroborates the interpretation in Keshner and Peterson (1995) that reflexes stabilize the head on the trunk in this frequency domain.

There are also several pitfalls present when doing statistical calculations based on an artificially split dataset as that seen in Figure 5, whether the analysis is applied to the entire dataset (in which case even a mean value may become meaningless) or one is misled to assume that the test population represents two qualitatively different groups and choose to analyze them separately. These problems do not arise when analyzing the head's angle with respect to the room. This preferred mode of analysis also has a simple pragmatic justification; keeping the head steady in space is the very task that the subjects were instructed to perform, and it thus seems reasonable to choose the head‐room angle as the primary quantity to be studied.

In conclusion, our method of analysis used in this study removes a notably ambiguous interpretation of the results. The original method, using the trunk as the frame of reference, may produce graphical illustrations particularly for phase that suggests different behavior in different groups of subjects and additionally may obscure the interpretation whether reflexes stabilize the head on the trunk. Using the room as the frame of reference removes an artifical 360^o^ phase split that falsely indicates different behavoiur in some subjects. Results using the room as the frame of reference corroborate previous studies, suggesting that reflexes contribute to stabilization of the head on the trunk between 1 and 2 Hz. Thus, the present method will prevent erroneous interpretations on the basis of this particular mathematical artifact.

## Conflict of Interest

None declared.

## Supporting information




**Video S1.** Test situation with actuated chair.

**Video S2.** Animation from real data of response to sinusoidal input signals in ascending frequency. Magnitudes are exaggerated for visualization and do not correspond to magnitudes of chair motion in the experiment.
